# Three-Dimensionally Cultured Jaw Periosteal Cells Attenuate Macrophage Activation of CD4^+^ T Cells and Inhibit Osteoclastogenesis

**DOI:** 10.3390/ijms25042355

**Published:** 2024-02-16

**Authors:** Fang He, Liuran Wang, Felix Umrath, Andreas Naros, Siegmar Reinert, Dorothea Alexander

**Affiliations:** 1 Department of Oral and Maxillofacial Surgery, University Hospital Tübingen, 72076 Tübingen, Germany; hefangyx@outlook.com (F.H.); liuran.wang@med.uni-tuebingen.de (L.W.); felix.umrath@med.uni-tuebingen.de (F.U.); andreas.naros@med.uni-tuebingen.de (A.N.); siegmar.reinert@med.uni-tuebingen.de (S.R.); 2 Clinic for Orthopaedic Surgery, University Hospital Tübingen, 72072 Tübingen, Germany

**Keywords:** jaw periosteal cells, mesenchymal stem cells, β-tricalcium phosphate scaffolds, THP-1, macrophage polarization, secretomes, CD4^+^ T cells, osteoclastogenesis, pit area resorption assay

## Abstract

The implementation of a successful therapeutic approach that includes tissue-engineered grafts requires detailed analyses of graft-immune cell interactions in order to predict possible immune reactions after implantation. The phenotypic plasticity of macrophages plays a central role in immune cell chemotaxis, inflammatory regulation and bone regeneration. The present study addresses effects emanating from JPC-seeded β-TCP constructs (3DJPCs) co-cultivated with THP-1 derived M1/M2 macrophages within a horizontal co-culture system. After five days of co-culture, macrophage phenotype and chemokine secretion were analyzed by flow cytometry, quantitative PCR and proteome arrays. The results showed that pro-inflammatory factors in M1 macrophages were inhibited by 3DJPCs, while anti-inflammatory factors were activated, possibly affected by the multiple chemokines secreted by 3D-cultured JPCs. In addition, osteoclast markers of polarized macrophages were inhibited by osteogenically induced 3DJPCs. Functional assays revealed a significantly lower percentage of proliferating CD4^+^ T cells in the groups treated with secretomes from M1/M2 macrophages previously co-cultured with 3DJPCs compared to controls without secretomes. Quantifications of pit area resorption assays showed evidence that supernatants from 3DJPCs co-cultured with M1/M2 macrophages were able to completely suppress osteoclast maturation, compared to the control group without secretomes. These findings demonstrate the ability of 3D cultured JPCs to modulate macrophage plasticity.

## 1. Introduction

Bone defects in oral and maxillofacial surgery can be caused by various reasons, such as infection, trauma and neoplastic lesions [1]. The critical bone defects and the resulting impairment of oral function are challenging to mitigate [2,3]. In recent years, tissue engineering has been recognized as a promising new approach for regenerating bone tissue defects in orthopedic and oral and maxillofacial surgery [4]. Cells, scaffolds and bioactive molecules constitute three basic elements of bone tissue engineering (BTE) and largely determine the success or failure of bone tissue engineering constructs [4]. Mesenchymal stem cells (MSCs) from various stromal tissues and adult organs are considered to be suitable cell sources for BTE [5]. Among them, jaw periosteum-derived mesenchymal stem cells (JPCs) represent an ideal stem cell source for BTE applications in oral and maxillofacial surgery and regenerative medicine because of their high osteogenic potential, good accessibility and immunomodulatory ability [6,7,8,9].

Macrophages defend against microbial infections and maintain tissue homeostasis. They play a key role in the innate and adaptive immune response and the repair of dam-aged tissues [10]. In general, macrophages are divided into classically activated macro-phages (M1) and alternatively activated macrophages (M2) [11]. M1 macrophages induced by lipopolysaccharide (LPS) and interferon-γ (IFN-γ) are able to promote the development of inflammation [11]. In contrast, anti-inflammatory cytokines (such as IL-4 and IL-13) induce the polarization of M2 macrophages which not only have tissue repair functions but also promote osteogenesis [11,12].

In our previous study, we demonstrated that two-dimensionally (2D)-cultured JPCs were able to regulate the polarization of THP-1-derived macrophages, shifting their phenotype from a pro-inflammatory to an anti-inflammatory phenotype in a horizontal co-culture system [6]. Compared to 2D cell culture systems, three-dimensional (3D) cell culture systems more realistically simulate the microenvironment in which cells occur in vivo, showing biological properties more similar to those of cells in a physiological context [13]. In particular, 3D cell culture conditions induce cell behaviors, such as secretion patterns, that more closely resemble in vivo conditions [14,15]. β-tricalcium phosphate (β-TCP) is generally considered as an ideal material for inorganic scaffolds in BTE and shows beneficial properties such as osteoconductivity, osteoinductivity and resorptive properties resulting in its susceptibility to degradation [16,17]. Therefore, the use of β-TCP scaffolds colonized by JPCs is a suitable approach to regenerate bone defects.

Based on the clinical background and our previous findings, the present study attempts to explore the effects of 3D cultured JPCs on macrophage polarization and plasticity. A horizontal co-culture system was used to co-culture the JPC-colonized β-TCP scaffolds (3DJPCs) and THP-1-derived macrophages in parallel chambers. M1/M2 macro-phage polarization was determined by examining cell surface markers and gene expression patterns. Paracrine crosstalk between 3DJPCs and macrophages was analyzed by chemokine secretion in the co-culture system. Functional assays should clarify to what extent 3D JPC constructs can influence the plasticity of THP-1 derived macrophages.

## 2. Results

### 2.1. Phenotypic Changes of THP-1-Derived M1/M2 Macrophages Co-Cultured with 3D-Cultured JPCs

The abbreviations for the experimental groups, containing the cell type and media conditions, are provided in Table 1, in order to facilitate the understanding of the terminology from the beginning.

**Figure 1:**
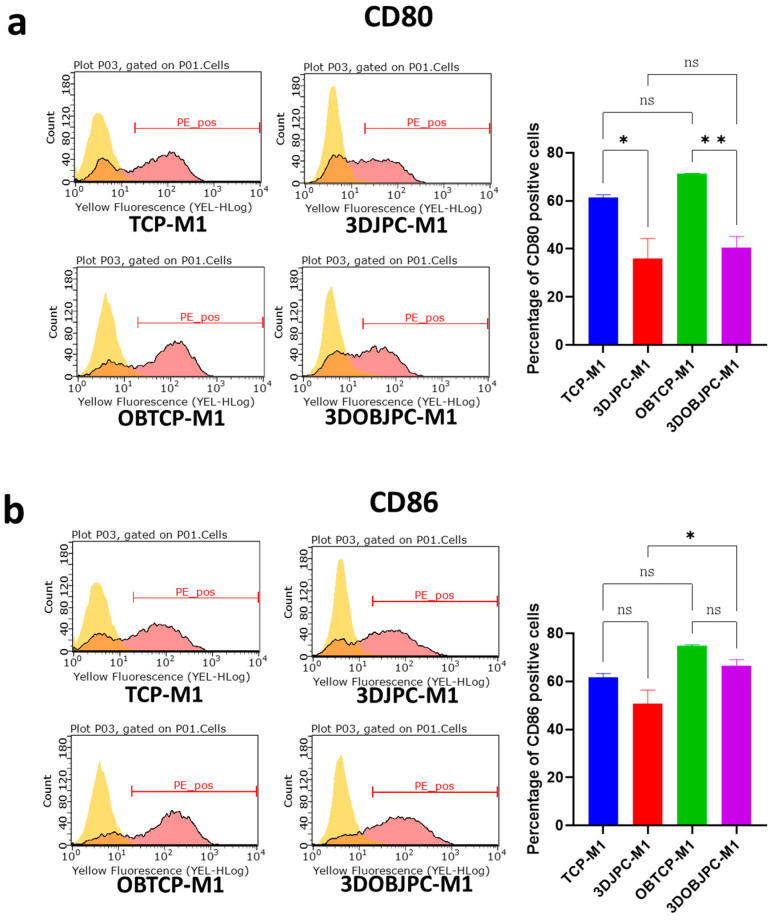
CD80 and CD86 surface marker expression of M1-macrophage co-cultures. Representative histograms (light yellow peaks: isotype control; red peaks: target markers) and graphs showing the CD80 (**a**) and CD86 (**b**) expression of M1 macrophages after five days of co-culture with 3DJPCs/3DOBJPCs, as detected by flow cytometry. Data are displayed as means ± SEM and analyzed by one-way ANOVA (n = 3, * = *p* < 0.05, ** = *p* < 0.01), ns = not significantly different.

After five days of co-culture with JPC-seeded scaffolds, the cell surface markers of M1/M2 macrophages were detected by flow cytometry (Figure 1). For co-cultured M1 macrophages, the percentage of CD80^+^ cells was significantly lower in the 3DJPCs/3DOBJPC-M1 co-culture groups compared to the TCP/OBTCP-M1 control groups (TCP-M1 61.42 ± 1.16 vs. 3DJPC-M1 36.13 ± 8.26, *p* < 0.05; OBTCP-M1 71.29 ± 0.14 vs. 3DOBJPC-M1 40.41 ± 4.85, *p* < 0.01). Similarly, the percentage of CD86^+^ cells was shown to be decreased in tendency (without reaching significance) in 3DJPC/3DOBJPC-M1 co-culture groups compared to the TCP/3DTCP-M1 control groups.

**Figure 2:**
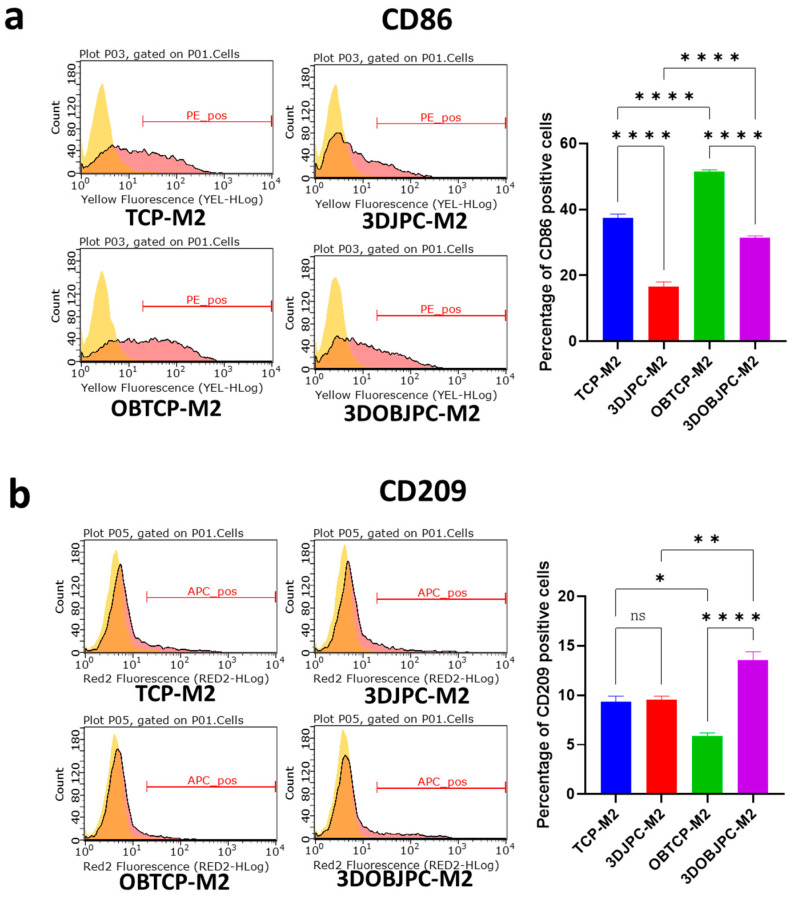
CD86 and CD209 surface marker expression of M2-macrophage co-cultures. Representative histograms (light yellow peaks: isotype control; red peaks: target markers) and graphs showing the percentage of the CD86 (**a**) and CD209 (**b**) positivity of M2 macrophages after five days of co-culture with 3DJPCs/3DOBJPCs, as detected by flow cytometry. Data are displayed as means ± SEM and analyzed by one-way ANOVA (n = 3, * = *p* < 0.05, ** = *p* < 0.01, **** = *p* < 0.0001), ns = not significantly different.

In contrast to M1-macrophages, co-cultured M2 macrophages showed significantly down-regulated CD86 surface marker expression in the 3DJPCs/3DOBJPCs-M2 co-culture groups when compared to the cell-free TCP/OBTCP-M2 control groups (TCP-M2 37.49 ± 1.15 vs. 3DJPC-M2 16.65 ± 1.40, *p* < 0.0001; OBTCP-M2 51.45 ± 0.52 vs. 3DOBJPC-M2 31.34 ± 0.61, *p* < 0.0001). Regarding CD209 surface marker expression, increased levels were detected in the 3DOBJPC-M2 co-culture group when compared with the cell-free OBTCP-M2 control group (OBTCP-M2 5.87 ± 0.34 vs. 3DOBJPC-M2 13.55 ± 0.86, *p* < 0.0001) (Figure 2).

### 2.2. Quantitative PCR Analysis of Polarization-Related Genes in M1/M2 Macrophages Co-Cultured with 3DJPCs/3DOBJPCs

**Figure 3:**
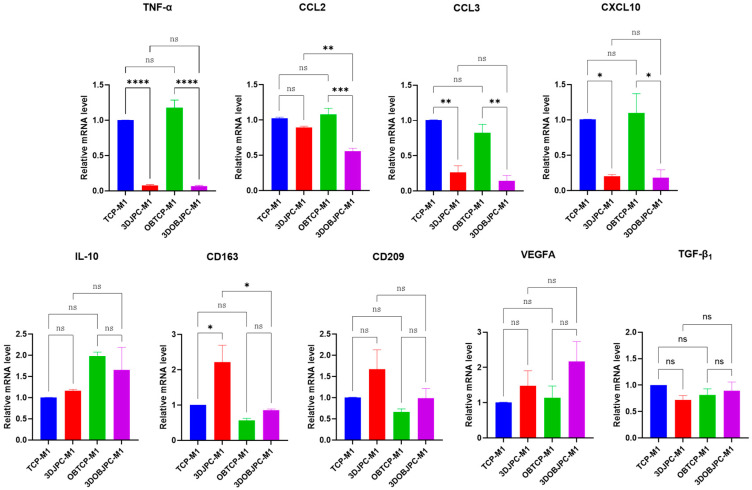
Gene expression of *TNF-α*, *CCL-2*, *CCL3*, *CXCL10*, *IL-10*, *CD163*, *CD209*, *VEGFA*, and *TGF-β*_1_ in M1 macrophages co-cultured for 5 days with 3DJPCs/OB3DJPCs (3DJPCs/OB3DJPCs-M1 groups) compared to M1 macrophages co-cultured with cell-free β-TCP scaffolds (TCP/OBTCP-M1 groups). Relative fold gene expression was calculated using the 2^−(∆∆Ct)^ method and mRNA levels in the TCP-M1 group were set as 1. Data are displayed as means ± SEM and analyzed by one-way ANOVA (n = 3, * = *p* < 0.05, ** = *p* < 0.01, *** = *p* < 0.001, **** = *p* < 0.0001), ns = not significantly different.

After five days of horizontal co-culture with 3DJPCs/3DOBJPCs, macrophage polarization-related genes in M1 and M2 macrophages were analyzed using quantitative PCR (Figure 3).

Co-cultured M1 macrophages showed significantly lower *TNF-α*, *CCL3*, and *CXCL10* gene expression levels in 3DJPC/3DOBJPC-M1 co-culture groups compared to the cell-free TCP/OBTCP-M1 control groups. Further, the gene expression levels of *CCL2* and *CXCL1* were significantly down-regulated in the 3DOBJPC-M1 co-culture group compared to the JPC-free OBTCP-M1 control group. *CD163* gene expression was significantly increased in the 3DJPC-M1 group compared to the JPC-free TCP-M1 group. *CD209* and *VEGFA* gene expression showed an upward trend in 3DJPC-M1 or 3DOBJPC-M1 co-culture groups without reaching significance. Obtained data are listed in Table 2.

**Figure 4:**
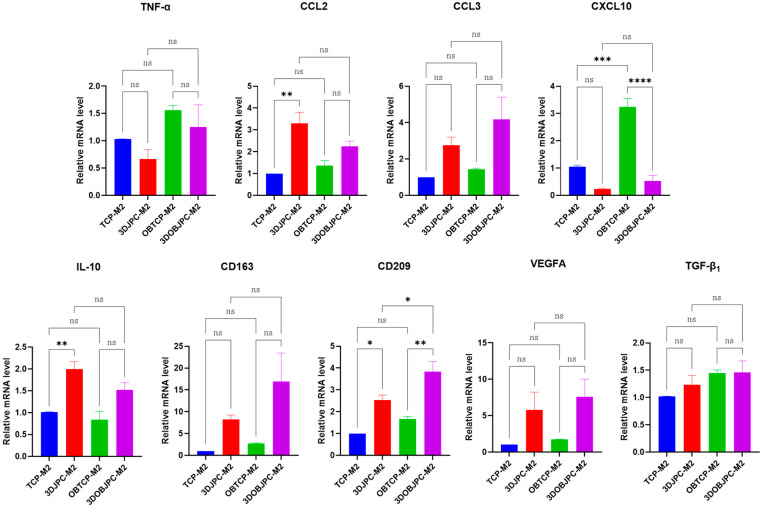
Gene expression of *TNF-α*, *CCL-2*, *CCL3*, *CXCL10*, *IL-10*, *CD163*, *CD209*, *VEGFA*, and *TGF-β*_1_ in M2 macrophages co-cultured for 5 days with untreated/osteogenically induced 3DJPCs (3DJPCs/3DOBJPCs-M2 groups) and cell-free β-TCP scaffold controls (TCP/OBTCP-M2 groups). Relative fold gene expression was calculated using the 2^−(∆∆Ct)^ method and mRNA levels in TCP-M2 were set to 1. Data are displayed as means ± SEM and analyzed by one-way ANOVA (n = 3, * = *p* < 0.05, ** = *p* < 0.01, *** = *p* < 0.001, **** = *p* < 0.0001), ns = not significantly different.

Figure 4 shows the gene expression patterns of M2 macrophages co-cultured with cell-free or JPC-seeded β-TCP constructs (obtained values are listed in Table 3). Co-cultured M2 macrophages showed significantly up-regulated *CCL2* and *IL-10* gene expression in the 3DJPC-M2 group when compared to the cell-free TCP-M2 control. In contrast, *CXCL10* was significantly down-regulated in the 3DOBJPC-M2 co-culture group when compared to the cell-free OBTCP-M2 group. *CD209* gene expression in the 3DJPC/3DOBJPC-M2 groups was significantly elevated when compared with that in the JPC-free TCP/OBTCP-M2 groups. The expression level of pro-inflammatory *TNF-α* in co-cultured M2 macrophages also showed a decreasing trend in the 3DJPC/3DOBJPC-M2 groups compared to the control groups. In contrast, the expression of *CCL3*, *CD163* and *VEGFA* genes showed an increasing trend in the 3DJPC/3DOBJPC-M2 groups when compared with the cell-free controls TCP/OBTCP-M2. *TGF-β*_1_ which is a marker for M2 macrophages was shown to be slightly induced by 3DJPCs but also by osteogenic conditions, without reaching significant differences between the groups. In contrast, Figure 3 shows the tendency of lower levels of *TGF-β*_1_ in M1 macrophages co-cultured with 3DJPCs.

### 2.3. Quantitative PCR Analysis of Osteoclastogenesis-Related Genes in M1/M2 Macrophages Co-Cultured with 3DJPCs/3DOBJPCs

**Figure 5:**
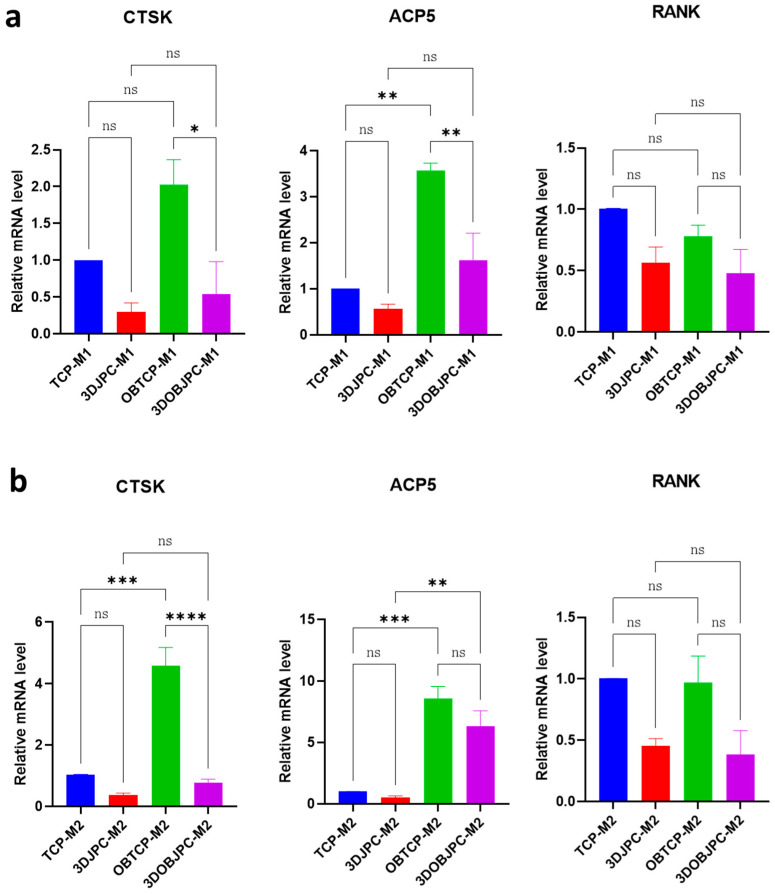
Expression of osteoclastogenesis-related genes (*CTSK*, *ACP5* and *RANK*) in M1/M2 macrophages co-cultured for 5 days with untreated/osteogenically induced 3DJPCs or cell-free β-TCP scaffolds. (**a**): Gene expression in co-cultured M1 macrophages. (**b**): Gene expression in co-cultured M2 macrophages. Relative fold gene expression was calculated using the 2^−(∆∆Ct)^ method and mRNA levels in TCP-M1 or TCP-M2 groups were set to 1. Data are displayed as means ± SEM and analyzed by one-way ANOVA (n = 3, * = *p* < 0.05, ** = *p* < 0.01, *** = *p* < 0.001, **** = *p* < 0.0001), ns = not significantly different.

After five days of co-culture, genes associated with osteoclastic differentiation (*CTSK* (cathepsin K), *ACP5* (tartrate-resistant acid phosphatase 5 or TRAP) and *TNFRSF11A* (TNF receptor superfamily member 11a or RANK)) were analyzed in M1/M2 macrophages by quantitative PCR (Figure 5). The gene expression levels of *CTSK* and *ACP5* were shown to be significantly lower in the 3DOBJPC-M1 group compared with levels detected in the JPC-free OBTCP-M1 group (*CTSK*: OBTCP-M1 2.02 ± 0.34 vs. 3DOBJPC-M1 0.54 ± 0.44, *p* < 0.05; *ACP5*: OBTCP-M1 3.57 ± 0.16 vs. 3DOBJPC-M1 1.62 ± 0.59, *p* < 0.01). Similarly, *CTSK* gene expression levels were significantly reduced in the 3DOBJPC-M2 group when compared to those detected in the OBTCP-M2 control group (*CTSK*: OBTCP-M2 4.58 ± 0.59 vs. 3DOBJPC-M2 0.76 ± 0.13, *p* < 0.0001). *RANK* gene expression in the 3DJPC/3DOBJPC-M1 or 3DJPC/3DOBJPC-M2 groups showed a down-regulation trend when compared with JPC-free TCP/OBTCP-M1 or TCP/OBTCP-M2 control groups without reaching significance.

### 2.4. Quantitative PCR Analysis of Osteogenesis and Immunomodulation-Related Genes in 3DJPCs/3DOBJPCs Co-Cultured with M1/M2 Macrophages

**Figure 6:**
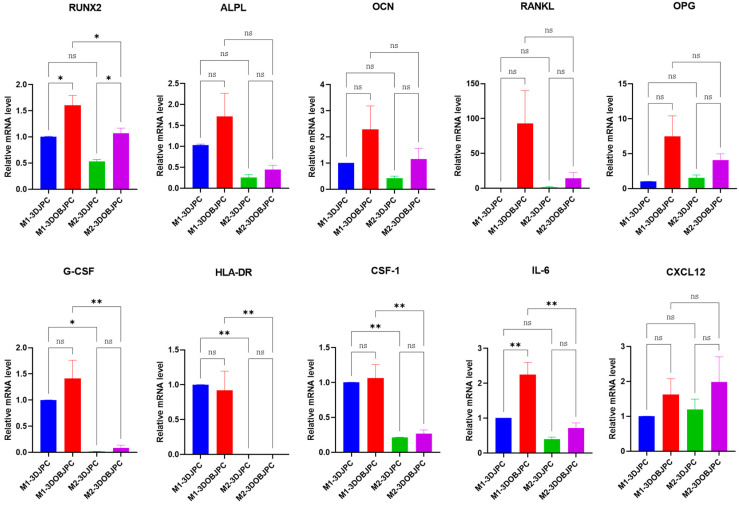
*RUNX2*, *ALPL*, *OCN*, *RANKL*, *OPG*, *G-CSF*, *HLA-DR*, *CSF-1*, *IL-6* and *CXCL12* gene expression of 3DJPCs/3DOBJPCs co-cultured for 5 days with M1/M2 macrophages analyzed by quantitative PCR. Relative fold gene expression was calculated using the 2^−(∆∆Ct)^ method and mRNA levels in the 3DJPC co-cultured with M1 macrophages (M1-3DJPC group) were set to 1. Data are displayed as means ± SEM and analyzed by one-way ANOVA (n = 3, * = *p* < 0.05, ** = *p* < 0.01), ns = not significantly different.

After five days of co-culture with M1/M2 macrophages, the expression of genes related to osteogenic differentiation and immunomodulation was analyzed in 3DJPCs treated with control and osteogenic medium (3DJPCs/3DOBJPCs) (Figure 6).

*RUNX2* gene expression levels were significantly up-regulated in 3DOBJPCs regardless of whether they were co-cultured with M1 or M2 macrophages (M1-3DOBJPC/M2-3DOBJPC groups) compared to the untreated groups M1-3DJPC/M2-3DJPC (M1-3DJPC 1.01 ± 0.01 vs. M1-3DOBJPC 1.60 ± 0.19, *p* < 0.05; M2-3DJPC 0.53 ± 0.04 vs. M2-3DOBJPC 1.07 ± 0.10, *p* < 0.05). Further, 3DOBJPCs showed an up-regulation trend in *ALPL*, *OCN*, *RANKL* and *OPG* genes expression compared to untreated 3DJPCs controls without reaching significant values.

The gene expression levels of immunomodulatory factors *G-CSF*, *HLA-DR* and *CSF-1* in 3DJPCs/3DOBJPCs co-cultured with M1 macrophages were significantly elevated compared to 3DJPCs/3DOBJPCs co-cultured with M2 macrophages (*G-CSF*: M1-3DJPC 1.00 ± 0.00 vs. M2-3DJPC 0.02 ± 0.01, *p* < 0.05, M1-3DOBJPC 1.41 ± 0.35 vs. M2-3DOBJPC 0.09 ± 0.05, *p* < 0.01; HLA-DR: M1-3DJPC 1.00 ± 0.00 vs. M2-3DJPC 0.00 ± 0.00, *p* < 0.01, M1-3DOBJPC 0.92 ± 0.28 vs. M2-3DOBJPC 0.00 ± 0.00, *p* < 0.01; *CSF-1*: M1-3DJPC 1.00 ± 0.00 vs. M2-3DJPC 0.21 ± 0.01, *p* < 0.01, M1-3DOBJPC 1.06 ± 0.19 vs. M2-3DOBJPC 0.27 ± 0.05, *p* < 0.01). *IL-6* gene expression in the M1-3DOBJPC group was shown to be significantly higher compared to the M1-3DJPC group (M1-3DJPC 1.00 ± 0.00 vs. M1-3DOBJPC 2.25 ± 0.35, *p* < 0.01) and compared to the M2-3DOBJP group.

### 2.5. Chemokine Secretion by M1/M2 Macrophages Co-Cultured with 3DJPCs/3DOBJPCs Ana-lyzed by Proteome Profiler Arrays

After five days of co-culture of 3DJPC/3DOBJPC and M1/M2 macrophages, chemokine secretions in supernatants collected from M1/M2 macrophage chambers were analyzed by using proteome profiler arrays.

**Figure 7:**
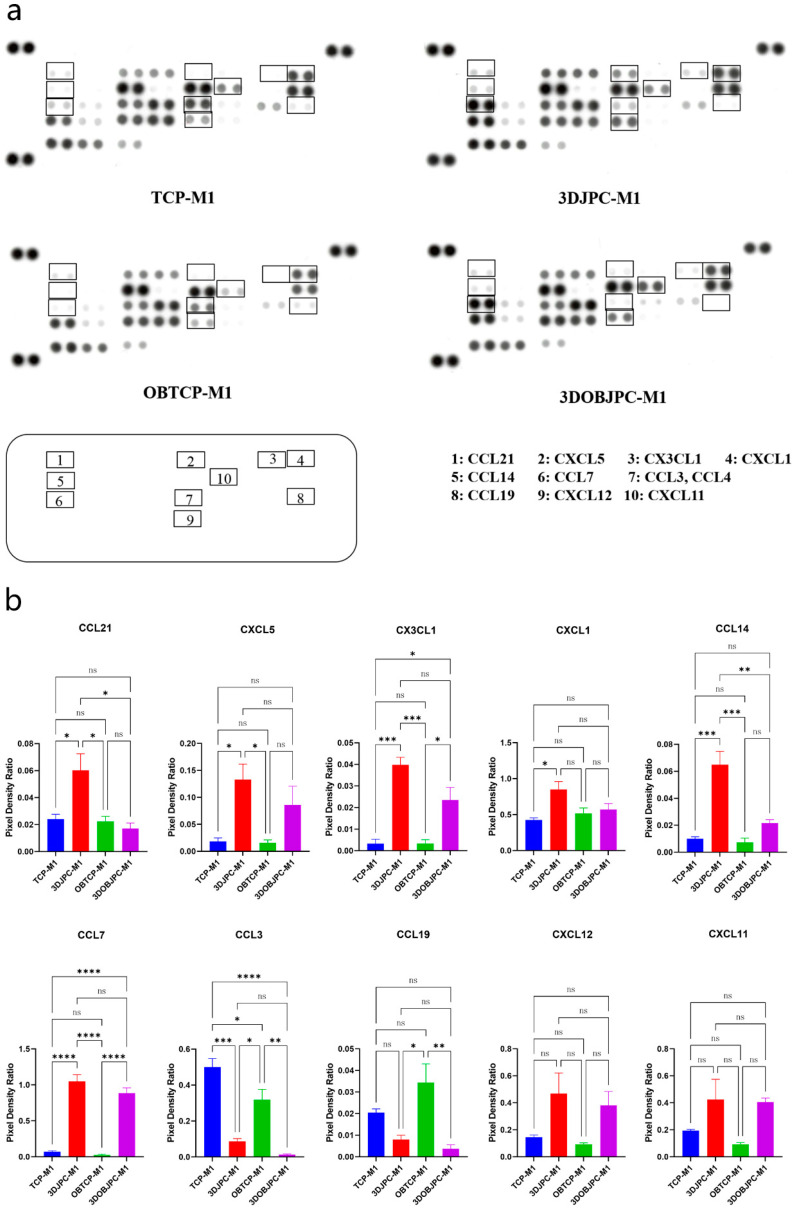
Chemokine secretion in supernatants of M1 macrophages co-cultured with 3DJPCs/3DOBJPCs was analyzed with proteome profiler arrays. (**a**): X-rays of representative membranes showing chemokine dot blots in different groups (rectangle: detected differential spots between groups). (**b**): Quantification of pixel densities by the ImageJ software for the chemokine dot blots marked in a. Data are displayed as means ± SEM and analyzed by one-way ANOVA (n = 3, * = *p* < 0.05, ** = *p* < 0.01, *** = *p* < 0.001, **** = *p* < 0.0001), ns = not significantly different.

Figure 7a shows the representative membranes of the four groups. The levels of CX3CL1 and CCL7 secretion detected in the supernatants collected from the 3DJPC-M1/3DOBJPC-M1 groups were significantly higher compared to those from TCP-M1/OBTCP-M1 groups. The secretion levels of CCL21, CXCL5, CXCL1 and CCL14 were significantly higher in the 3DJPC-M1 group when compared to the JPC-free TCP-M1 control group. In contrast, CCL3/CCL4 levels in the 3DJPC-M1/3DOBJPC-M1 groups were significantly reduced compared with those in the TCP-M1/OBTCP-M1 groups. CCL19 secretion levels were significantly decreased in the 3DOBJPC-M1 group compared to those of the JPC-free OBTCP-M1 group. Compared to the TCP-M1/OBTCP-M1 groups, CXCL12 and CXCL11 showed elevated trends in the 3DJPC-M1/3DOBJPC-M1 groups without reaching significance (Figure 7b and Table 4).

**Figure 8:**
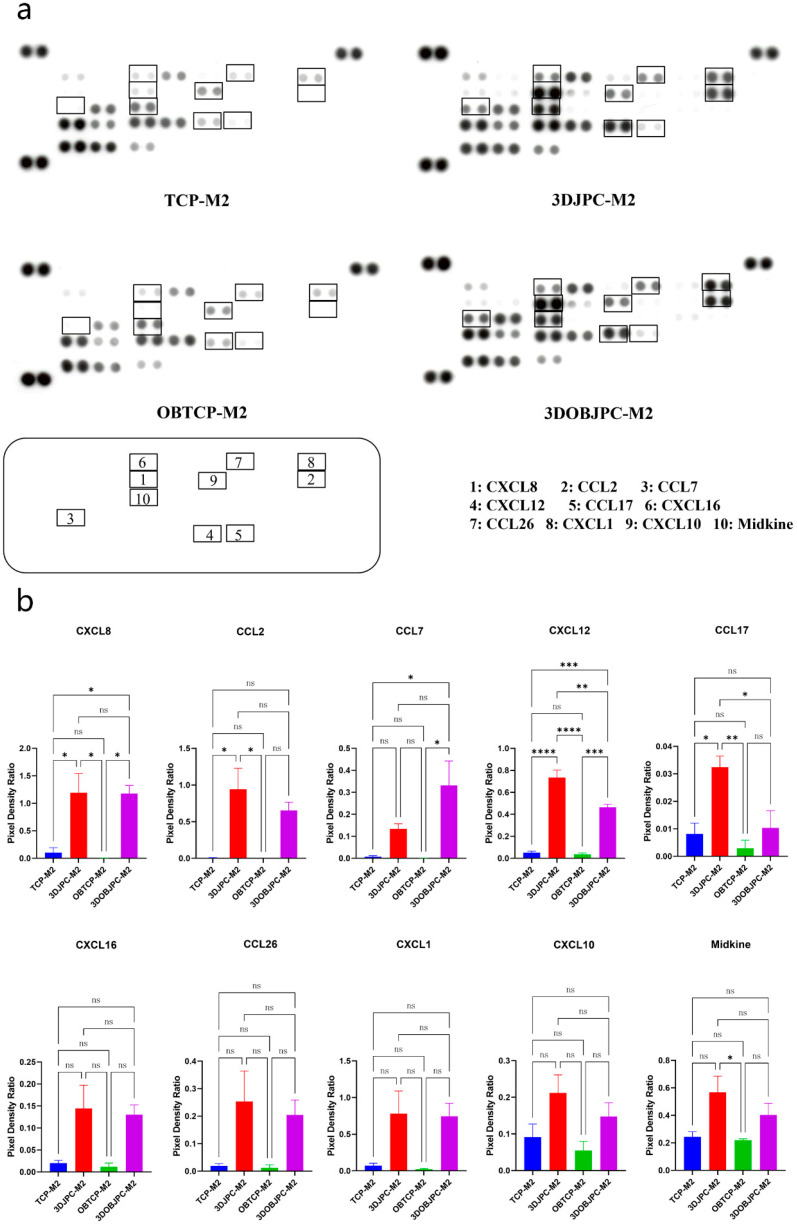
Chemokine secretion in supernatants of M2 macrophages co-cultured with 3DJPCs/3DOBJPCs was analyzed with proteome profiler arrays. (**a**): X-rays of representative membranes with chemokine dot blots in different groups (rectangle: detected differential spots between groups). (**b**): Quantification of pixel densities by the ImageJ software for chemokine dot blots marked in a. Data are displayed as means ± SEM and analyzed by one-way ANOVA (n = 3, * = *p* < 0.05, ** = *p* < 0.01, *** = *p* < 0.001, **** = *p* < 0.0001), ns = not significantly different.

In supernatants from M2 macrophages, CXCL8 and CXCL12 were shown to be significantly higher in the 3DJPC-M2/3DOBJPC-M2 groups compared to the TCP-M2/OBTCP-M2 groups. The secretion of CCL2 and CCL17 was significantly higher in the 3DJPC-M2 group compared with the JPC-free TCP-M2 group. CCL7 secretion was significantly up-regulated in the 3DOBJPC-M2 group when compared to the JPC-free OBTCP-M2 group. In addition, CXCL16, CCL26, CXCL1, CXCL10 and Midkine levels showed an increasing trend in the 3DJPC-M2/3DOBJPC-M2 groups compared to the TCP-M2/OBTCP-M2 control groups without reaching significance (Figure 8 and Table 5).

### 2.6. Functional Assay: Effects of M1/M2 Macrophage Secretomes from Co-Cultures with 3DJPCs/3DOBJPCs on the Proliferation Activity of CD4^+^ T Cells

To investigate the effects of M1/M2 macrophages in the CD4^+^ T cell regulation, we involved the secretomes obtained from the M1/M2 macrophages chambers co-cultured with 3DJPCs/3DOBJPCs for 5 days in the T cell proliferation process for a duration of 3 days. It must be kept on mind that secretomes collected from macrophage/JPC chambers do not contain exclusively macrophage-/JPC-secreted factors, but also those from the co-cultured cell type, based on the fact that particle exchange is quite effective in the horizontal co-culture plate, as previously published [6].

**Figure 9:**
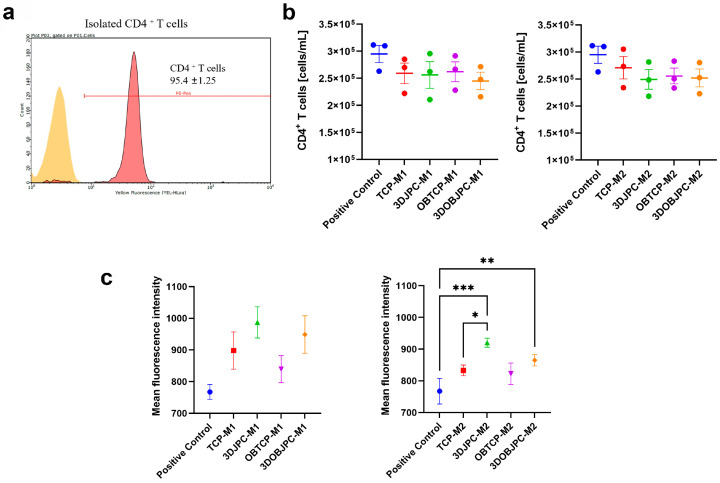
Effects of M1/M2 secretomes on the CD4^+^ T cell proliferation ability. (**a**): Purity of CD4^+^ T cells after isolation using the CD4^+^ T cell isolation kit (%). (**b**): Analysis of CD4^+^ T cell concentration (cells/mL). (**c**): Mean fluorescence intensity of proliferating CD4^+^ T cells. Data are displayed as means ± SEM and analyzed by one-way ANOVA (n = 3, * = *p* < 0.05, ** = *p* < 0.01, *** = *p* < 0.001).

The experimental results demonstrated a reduction in the number of CD4^+^ T cells in the presence of M1/M2 secretomes compared to the positive control group without secretomes. The number of proliferating T cells in the 3DOBJPC-M1 group was decreased compared to the 3DJPC-M1 group (Figure 9b, left panel) and compared to the positive control but without reaching significance. Further, lower numbers of proliferating CD4^+^ T cells were detected in the 3DJPC-M2/3DOBJPC-M2 secretome groups compared to the TCP-M2/OBTCP-M2 medium groups and the positive control, also without reaching significance (Figure 9b, right panel). Reduced fluorescence intensities correlate with a higher cell proliferation because the greater the number of cell divisions, the lower will be the measured CFSE fluorescence intensities. The 3DJPC-M1/3DOBJPC-M1 groups exhibited higher mean fluorescence intensities compared to those detected in the medium groups (TCP-M1/OBTCP-M1), and similar results were obtained in the 3DJPC-M2/3DOBJPC-M2 groups compared to the respective medium controls (TCP-M2/OBTCP-M2) reaching significances compared to the positive control (Figure 9c) and in the 3DJPC-M2 group compared to the respective TCP-M2 control group. These results indicate that the lowest proliferative activities of CD4^+^ T cells were detected in the groups incubated in the presence of M1/M2 secretomes obtained from 3DJPC/3DOBJPC co-cultures.

### 2.7. Functional Assay: Effects of 3DJPCs/3DOBJPCs Secretomes Collected from Co-Cultures with M1/M2 Macrophages on the Differentiation of Osteoclasts

In order to evaluate the effects emanating from 3DJPC secretomes collected after co-culturing with M1/M2 macrophages on the maturation of osteoclasts, pit area resorption assays were performed. As mentioned in the chapter before, the collected secretomes represent a mixture of factors secreted by both co-cultured cell types, due to mutual influences through the diffusion of particles.

**Figure 10:**
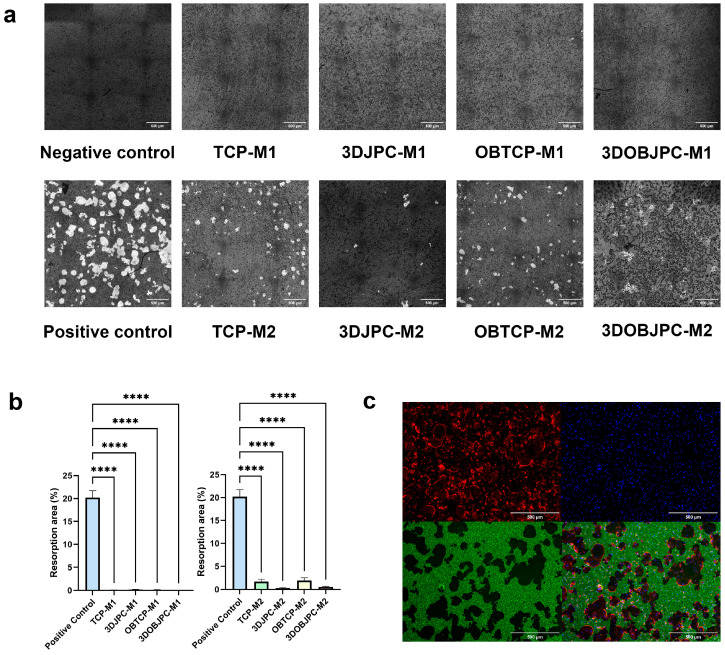
Effects of secretomes from JPC chambers after co-culturing with M1/M2 macrophages on osteoclast differentiation of PBMCs. PBMCs were seeded on CaP coated plates and incubated with JPC-secretomes from different M1/M2 co-culture conditions for 6 days. (**a**): CaP coating was stained by AgNO_3_ to visualize osteoclastic resorption pits. (**b**): Quantification of pit area was performed using the ImageJ software. Data are displayed as means ± SEM and analyzed by one-way ANOVA (n = 3, **** = *p* < 0.0001). (**c**): Representative image of PBMCs after 6 days of osteoclast differentiation (positive control). The cells were stained for actin and nuclei by phalloidin (red fluorescence) and Hoechst (blue fluorescence) to detect multinucleated osteoclasts with actin rings. CaP coating was stained by calcein (green fluorescence), and resorption pits are visible as black areas. Scale bar = 500 µm.

For the osteoclast differentiation analyses, osteoclasts were cultured on CaP coated plates. In order to visualize the positive controls, cells were stained by phalloidin, so actin ring formation, which is essential for the osteoclastogenic resorption, could be detected. Cell nuclei were stained by Hoechst (blue color), calcein was used for CaP visualization (green color) and resorption pit areas were visible as black areas (Figure 10c). For the quantification of resorption pit areas, CaP coating was stained by AgNO_3_ and resorbed pits were visible as bright spots (Figure 10a). Part b of Figure 10 clearly demonstrates that secretomes derived from 3DJPCs/3DOBJPCs chambers co-cultured with M1 macrophages reduced osteoclast formation completely compared to the positive control. Further, secretomes collected from 3DJPCs/3DOBJPCs chambers, previously co-cultured with M2 macrophages, exhibited a pronounced suppressive effect on both osteoclast formation and resorption activity. This suggested a potential regulatory role of secreted factors by 3DJPCs in modulating osteoclast development and function.

## 3. Discussion

In the field of regenerative medicine, strategies must be developed in order to transform inert synthetic biomaterials into smart implants. The immune response caused by tissue engineering products is multifactorial, and recent studies highlight the importance of fine-tuning the balance between pro- and anti-inflammatory reactions to support endogenous defect regeneration [18]. In this context, macrophages play a crucial role in orchestrating inflammatory reactions and healing processes. The switch between pro- and anti-inflammatory functions occurs through the polarization of M1 towards M2 macrophages, which show specific characteristics, such as different cell surface markers and the secretion of different cytokines and chemokines [19].

In our study, flow cytometry results showed that CD80 (classically activated macro-phage marker) cell surface expression of M1 macrophages was significantly down-regulated by the co-culture of 3DJPCs and 3DOBJPCs (as shown in Figure 1), while *CD209* (alternatively activated macrophage marker) expression on the cell surface of M2 macrophages was significantly up-regulated (as shown in Figure 2) in the 3DOBJPCs co-culture group and as confirmed also on gene expression level [20,21]. The gene expression analyses of M1 macrophages revealed that monocyte chemotactic protein-1 (*MCP-1/CCL2*), macrophage inflammatory protein-1α (*MIP-1α/CCL3*), *CXCL1* and *CXCL10*, also known as interferon gamma-induced protein 10 (*IP-10*), were significantly down-regulated by 3DJPCs, as illustrated in Figure 3. The gene expression levels of anti-inflammatory markers such as *CD163* and *CD209*, which are typically expressed by M2 macrophages, were increased in co-cultured M1 macrophages by the influence of 3DJPCs.

After co-cultivation of M2 macrophages with 3DJPCs, the gene expression of pro-inflammatory factors, such as *TNF-α* and *CXCL10*, was decreased in tendency in M2 macrophages reaching significance only for *CXCL10* (as shown in Figure 4). Interestingly, *CCL2* and *CCL3* gene expression were increased in co-cultured M2 macrophages without achieving significant differences except in the 3DJPC group for *CCL2*, indicating the slight recruitment of immune cells, as already described for these chemokines [22]. *CD163*, *CD209*, *IL-10* and *VEGFA* gene expression levels in M2 macrophages were shown to be up-regulated when co-cultured with 3DJPC/3DOBJPC, reaching significant values for *CD209* expression and for IL-10 in the group co-cultured with untreated 3DJPCs. Summarizing this part of the results, a polarization towards the activation of the alternative path (M2 macrophages) becomes clearer and is in line with data already published in this context [23,24,25,26]. The herein observed effects emanating from 3DJPCs/3DOBJPCs constructs on macrophage plasticity are only noticeable in co-cultured M1/M2 cell groups, but not in PMA-induced MΦ macrophages. The lack of significance in some M1/M2 specific markers is probably due to the use of primary cells (JPCs) and secondly to the use of an immortalized cell line which differs in sensitivity and responsiveness to activator substances, compared to primary cells. Therefore, we tested the functionality of in co-cultures generated THP-1 derived M1/M2 macrophages by analyzing the effects of their secretomes on T cell proliferation. The proven efficacy shown in Figure 9 by reaching significances for M2 macrophages with exactly the same tendency for M1 macrophages, counterbalances their relative weak distinctive character.

The gene expression analysis of osteoclastogenic markers such as *CTSK* (cathepsin K) and *ACP5* (tartrate-resistant acid phosphatase type 5, TRAP) [27,28] in M1/M2 macro-phages showed partially significantly increased levels under osteogenic conditions when macrophages were co-cultured with JPC-free β-TCP scaffolds. This at first glance surprising result is understandable in so far as *CTSK* and *ACP5* are both enzymes with optimal activities under acidic conditions (osteogenic medium contains ascorbic acid) and as cell-free scaffolds are directly exposed to them, triggering scaffold surface degradation. Seeded cells cover the construct surface resulting in no direct exposition to the culture medium and therefore JPCs seemed to completely abolish *CTSK* and partially abolish ACP5 levels in macrophages under osteogenic conditions. Further, the mRNA levels of *TNFRSF11A (RANK)* were down-regulated in macrophages co-cultured with JPCs without reaching significance. RANK is the receptor for RANKL, which seemed to be slightly but not significantly induced, as shown in Figure 6 by osteogenically treated JPCs. These results indicate that co-cultivation with JPCs under osteogenic conditions does not effectively activate the osteoclastic differentiation of M1/M2 macrophages. Therefore, while JPCs shift macrophage polarization into the alternative path, they do not activate the differentiation of the osteoclastic lineage of macrophages at the same time, further contributing to bone formation. This result was clearly validated by the functional pit assay demonstrating the lack of pit formation in the presence of 3DJPC secretomes in comparison to the analyzed control group of osteoclasts without secretomes. For these analyses, we induced the osteoclast formation of PBMC-derived monocytes, since THP-1 capacity to differentiate into osteoclasts was relatively weak and not convincing.

Since MSCs show high plasticity, different studies could demonstrate that the priming of MSCs, in particular, under pro-inflammatory conditions, induces an enhanced immuno-modulatory MSC phenotype [29,30,31]. Our previous studies have repeatedly confirmed that the immunogenic, anti-inflammatory, angiogenic and osteogenic potentials of differentiated and undifferentiated JPCs vary and this finding explains the detected differences between the 3DJPC and 3DOBJPC groups.

Interestingly, analyses of osteogenesis-related genes such as *RUNX2*, *alkaline phosphatase (ALPL)*, *osteocalcin (OCN)* and *osteoprotegerin (OPG)* by JPCs in our study revealed the tendency of increased levels under pro- (LPS/IFN-γ) compared to anti-inflammatory (IL-4/IL-13) co-culture conditions, as illustrated in Figure 6. This result indicates that pro-inflammatory conditions seem to activate more effectively the beginning osteogenic differentiation process of 3DJPCs. On the other site, 3DJPCs up-regulated significantly their gene expression of *G-CSF*, *HLA-DR* and *CSF-1* genes under pro- compared to anti-inflammatory co-culture conditions. These chemokines have the ability to attract and stimulate leukocytes, play a central role in the homeostasis of the immune system and are involved in all protective or destructive immunoreactions to mediate both defense and tissue repair [32].

In our study, under pro-inflammatory conditions (IFN-γ/LPS), JPCs induced the secretion of CXCL5, CX3CL1, CXCL1, CCL14, CCL7, CXCL12 and CXCL11 in co-cultured M1 macrophages (Figure 7 and Table S1). Osteogenically activated JPCs completely abolished CCL3 and CCL19 secretion and significantly increased the secretion of CX3CL1 and CCL14 in the same cells, as illustrated in Figure 7b. Xuan and co-authors examined the distinct chemotaxis of M1 and M2 macrophages by different chemokines [33]. They found that among others, CCL3 and CCL19 specifically induced the chemotaxis of M1 macrophages, whereas CCL7 induced the chemotaxis of both M1 and M2 macrophages. Considering these findings, we hypothesize that the inhibition of the M1-specific chemokines CCL3 and CCL19 could contribute to the M1→M2 shift induced by JPCs. In addition, in the mentioned study by Xuan et al. [33], CXCL12 (stromal cell-derived factor-1, SDF-1) showed a trend of activating M1 macrophages more effectively, without reaching significance. In our study, the detected SDF-1 secretion is primarily attributable to JPCs, as demonstrated by the elevated levels in the supernatants of JPC monocultures (Figure S1 and previous data). In a recent work by Giri and colleagues, evidence showed that bone marrow MSCs secreted CCL2 form heterodimers with CXCL12 [34]. These heterodimers up-regulated IL-10 expression in CCR2^+^ macrophages in vitro. Further, CCL2 expression by MSCs was shown to be required for the IL-10^+^ M2 polarization of resident macrophages in vivo. Since we could show that co-cultured JPCs significantly up-regulated CCL2 secretion in M2 macrophages as well as CXCL12 in both M1 and M2 macrophages, and the receptor for the CCL2/CXCL12 heterodimers CCR2 was shown to be expressed at significantly higher levels on M1 macrophages, as published by Xuan et al. [33], we put forth the hypothesis that the CCL2/CXCL12 heterodimers released by JPCs are involved in the M1→M2 shift of THP-1 derived macrophages. CCL3 was shown to be a main stimulator of osteoclastogenesis. Jordan and co-authors could demonstrate that the inhibition of CCL3 abrogated precursor cell fusion and bone erosions in human osteoclast cultures [35]. Based on this finding, we speculate that the abrogation of CCL3 secretion by JPCs is involved in the JPC-mediated inhibition of osteoclastic genes in THP-1 macrophages, as shown in Figure 5.

Lin and co-authors could show evidence that secreted CXCL8 contributes to the development of an immunosuppressive microenvironment by inducing PD-L1 (programmed cell death 1 ligand 1) positivity of macrophages [36]. PD-L1 binds to B7.1 in cis on the same cell, preventing B7.1 from activating T cells via CD28 binding [37]. Lin et al. [36] further demonstrated that high levels of CXCL8+ macrophages was positively correlated with poor prognosis in patients with gastric cancer and concluded that the administration of CXCL8 inhibitors may result in an effective antitumor response. Based on these discoveries, we assume that JPC-secreted high levels of CXCL8 (Figure S1) induce the PD-L1 positivity of M1/M2 macrophages inhibiting interaction with T-cells.

In our study, the CXCR3 ligand, CXCL10, was secreted by macrophages and, in particular, by LPS/IFN-γ-stimulated 3D-cultured JPC in monocultures, as illustrated in Figure S1. The same figure reveals CXCL1 and CXCL10 detection solely in medium without cells (TCP-RPMI group), probably deriving from included FCS. When 3DJPCs are cultivated in RPMI, CXCL1 levels are slightly induced and CXCL10 levels are up-regulated under M1-inducing conditions. The levels of JPC-secreted CXCL10 under M2 conditions show similar levels as under RPMI conditions, CXCL1 levels are slightly induced compared to the cell-free sample. By gene expression analyses, *CXCL10* mRNA levels were significantly decreased in M1/M2 macrophages when co-cultured with osteogenically induced JPCs. That indicates that in co-cultures, secreted CXCL1 and CXCL10 levels originate from the FCS included in the RPMI medium and JPCs. However, the totality of JPC secreted factors is able to down-regulate the expression of both factors in M1/M2 macrophages.

Xuan et al. [33] reported on higher levels of CXCR3 on M1 compared to M2 macrophages, and a recent work by Pandey and co-authors reports on the highest CXCR3 levels in unpolarized macrophages with significantly reduced levels in M1 to nearly undetectable in M2 macrophages [38]. Due to this, M1 macrophages could be much more receptive to CXCL10 and thereby mediate pro-inflammatory responses [39,40,41].

**Figure 11:**
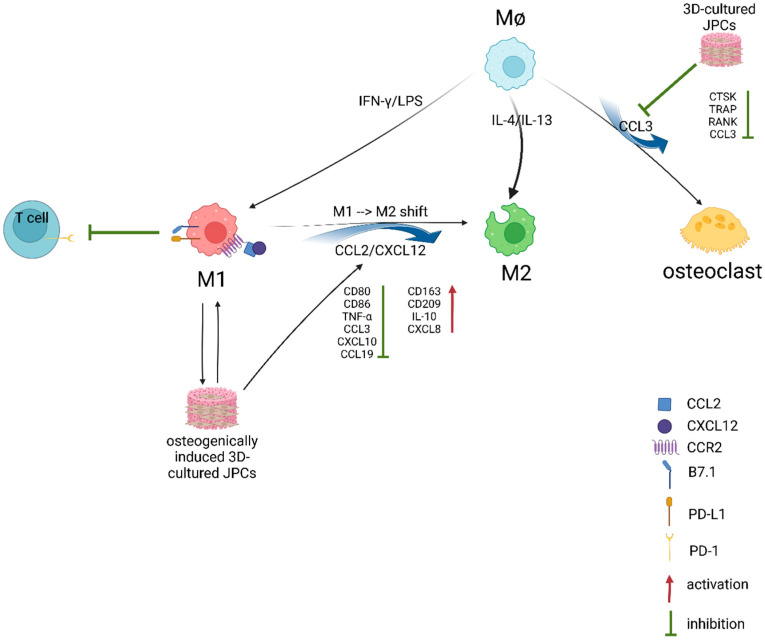
Summary of the results obtained in the present study. An overview of activating/inhibiting interactions between 3DJPCs and macrophages including the involved factors is provided. This illustration was created with BioRender.com, accessed on 20 October 2022.

We can assume that JPCs growing and differentiating within β-TCP constructs secrete a plethora of cyto- and chemokines involved in the initiation of immune responses. In a first step, JPC-secreted factors attract different immune cells to the site of implantation. In the following step, attracted cells react and also produce cyto- and chemokines by themselves. Summarizing the results of our study and considering our functional assays, we can draw the following conclusions and hypotheses (Figure 11). JPCs growing within β-TCP constructs inhibit osteoclastic differentiation of macrophages and attenuate the interaction of M1 macrophages with CD4^+^ T cells. On the other site, M1 macrophages induce the beginning osteogenesis of JPCs growing within β-TCP constructs. Further, osteogenically induced 3D-cultured JPCs induce an M1→M2 shift by the down-regulation of M1-specific cell surface markers, cyto- and chemokines resulting in the modulation of a rather M2-specific phenotype.

## 4. Materials and Methods

### 4.1. THP-1 and JPC Cell Culture

THP-1 cells were obtained from the American Type Culture Collection and expanded in RPMI 1640 medium (Thermo Fisher Scientific, Waltham, MA, USA) containing 10% heat-inactivated fetal bovine serum (FBS) (Sigma-Aldrich, Darmstadt, Germany), 1% penicillin/streptomycin (Lonza, Basel, Switzerland), 1% amphotericin B (Biochrom AG, Berlin, Germany) and 0.05 nM 2-mercaptoethanol (Sigma-Aldrich, Darmstadt, Germany).

After approval by the local ethics committee (No. 618/2017BO2), JPCs from three donors were included in this study. JPCs were cultured and expanded with DMEM/F12 medium (Thermo Fisher Scientific, Waltham, MA, USA) containing 5% hPL (provided by the Institute for Clinical and Experimental Transfusion Medicine of the University Hospital Tübingen), 1% penicillin/streptomycin (Lonza, Basel, Switzerland) and 1% amphotericin B (Biochrom AG, Berlin, Germany). Cell passaging of JPCs was performed with TrypLE-Express (Thermo Fisher Scientific, Waltham, MA, USA). Passage 4 of JPCs was used to culture on β-TCP scaffolds (Curasan AG, Kleinostheim, Germany). Details concerning the fabrication process are provided in the previous publication [42].

### 4.2. Preparation of JPC-Seeded β-TCP Scaffolds (3DJPCs) and Osteogenic Differentiation of 3DJPCs (3DOBJPCs)

3D cultivation of JPCs (3DJPCs) was achieved by seeding of JPCs on β-TCP scaffolds. First, β-TCP scaffolds were soaked in 5% hPL DMEM/F12 medium for 1 h in 96-well polypropylene plates. Next, 50 µL of cell suspension (5 × 10^4^ JPCs) were applied to each scaffold. After 2 h of incubation, an additional 150 µL of the medium was added to each scaffold. The following day, the JPC-seeded scaffolds were transferred to new 96-well plates containing 200 µL of medium per well for further cultivation.

After transferring 3DJPCs to new 96-well plates on day 1, the constructs were further cultured for 10 days either with DMEM/F12 + 10% hPL + 1% penicillin/streptomycin + 1% amphotericin B (untreated control) or with DMEM/F12 + 10% hPL + 1% penicillin/streptomycin + 1% amphotericin B + 100 μM L-ascorbic acid + 10 mM β-glycerophosphate (osteogenic condition). For cell-free controls, β-TCP scaffolds were incubated with control medium and osteogenic medium for 10 days in parallel 96-well plates. On day 11, scaffolds were transferred to chamber A of the co-culture system.

### 4.3. Co-Culture of 3DJPCs/3DOBJPCs and M1/M2 Macrophages

**Figure 12:**
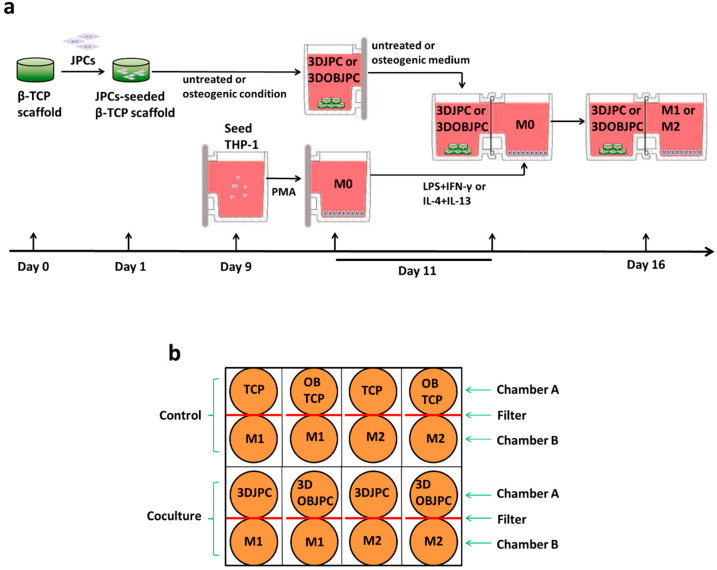
The co-culture procedure and the examined groups. (**a**): experimental flow chart of pre-culture and co-culture of 3DJPCs/3DOBJPCs and M1/M2 macrophage groups. (**b**): experimental setup of co-culture and control groups.

UniWells^TM^ Horizontal Co-culture Plates (Ginreilab Inc., Uchinada, Japan), containing filters (0.6 μm) and adapters (96-well plate size) were used for the co-culture of 3DJPCs/3DOBJPCs and THP-1 derived M1/M2 macrophages. This system allows separating the two chambers of the system to first culture cells individually and later join them together for co-culture. Figure 12 shows the experimental procedure of 3DJPC/3DOBJPC and M1/M2 macrophage co-cultures (a) and the experimental grouping of co-cultures (b) The abbreviations and groupings of the 3DJPCs/3DOBJPCs are shown in Table 1, shown in the Results section.

Macrophage differentiation of THP-1 cells started on day 9 of JPC treatment (Figure 12a). For M0 differentiation, THP-1 cells of passage 13 (4 × 10^5^) were re-suspended in 1.5 mL of RPMI 1640 medium + 5% hPL + 5 nM of phorbol 12-myristate 13-acetate (PMA, Sigma-Aldrich, Darmstadt, Germany) and then added to chamber B. The M0 macrophage induction lasted 48h (until day 11 of JPC treatment).

On day 11, co-cultures of 3DJPCs/3DOBJPCs and M1/M2 macrophages were set up by assembling chambers A and B. In the co-culture group, five constructs of 3DJPCs/3DOBJPCs were transferred into chamber A containing 1.3 mL of control or osteogenic medium. In chamber B, M0 macrophages were stimulated for M1 polarization with 1.5 mL of RPMI1640 medium + 5% hPL + 15 ng/mL lipopolysaccharide (LPS, Sigma-Aldrich, Darmstadt, Germany) + 20 ng/mL interferon-γ (IFN-γ, Sigma-Aldrich, Darmstadt, Germany). For the induction of M2 macrophages, 1.5 mL of RPMI1640 medium + 5% hPL + 20 ng/mL interleukin 4 (IL-4, Sigma-Aldrich, Darmstadt, Germany) + 20 ng/mL interleukin 13 (IL-13, Sigma-Aldrich, Darmstadt, Germany) were added to the cells (Figure 12a).

After assembling co-culture chambers A and B, each pair of co-culture plates was placed in a 96-well plate size adapter and cultured for a further five days. On day 16, flow cytometry analyses and gene expression measurements were performed for M1 or M2 macrophages in the 3DJPCs/3DOBJPCs + M1/M2 co-culture groups and the TCP/OBTCP + M1/M2 control groups. Additionally, supernatants from macrophage chambers were collected and used for proteome analysis using antibody arrays.

Monoculture experiments of 3DJPCswere conducted to assess the chemokine secretion by JPCs. Therefore, 3DJPCs with control medium in chamber A and cell-free chamber B containing RPMI1640 medium + 5% hPL were set as the untreated 3DJPCs-RPMI group (Figure S1a). For 3DJPC-M1RPMI control group and 3DJPC-M2RPMI control group, cell-free chamber B was filled with RPMI1640 medium + 5% hPL + LPS/IFN-γ and RPMI1640 medium + 5% hPL + IL-4/IL-13, respectively. JPC-free β-TCP scaffolds with control medium in chamber A and cell-free chamber B containing RPMI1640 medium + 5% hPL were set as TCP-RPMI control group. After five days of culture, the chemokine levels in chamber B were analyzed using the human chemokine proteome profiler arrays (Figure S1).

### 4.4. Detection of Surface Markers on M1/M2 Macrophages by Flow Cytometry

After co-culture of 3DJPCs/3DOBJPCs and M1/M2 macrophages for five days, macrophages were detached by using TrypLE-Express and the expression of cell surface markers of M1 or M2 macrophages was analyzed by flow cytometry. After centrifugation (1400 rpm, 5 min) of the collected macrophage suspension and removal of the supernatant, cell pellets were incubated with 10% Gamunex (human immune globulin solution, Talecris Biotherapeutics GmbH, Frankfurt, Germany) on ice. The cells were then incubated with fluorophor labeled CD80-PE, CD86-PE, and CD209-APC antibodies (Biolegend, San Diego, CA, USA) for 30 min in the dark. Subsequently, cells were washed twice with FACS buffer (PBS containing 0.1% BSA and 0.1% sodium azide), and surface marker expression was measured using a Guava EasyCyte 6HT-2L flow cytometer (Merck Millipore, Darmstadt, Germany). For data evaluation, guavaSoft 2.7 (EMD Millipore Corporation, Hayward, CA, USA) was used.

### 4.5. Quantitative PCR for Macrophage and 3DJPCs Gene Expression Analysis

After five days of co-culture of 3DJPCs/3DOBJPCs and M1/M2 macrophages, quantitative real-time PCR was used to analyze gene expression levels in co-cultured M1/M2 macrophages (groupings: TCP/OBTCP-M1/M2 and JPC/OBJPC-M1/M2) or JPCs/OBJPCs (groupings: M1/M2-JPC/OBJPC). Total RNA from macrophages was extracted using the NucleoSpin RNA kit (Macherey-Nagel, Hoerd, France). After quantification with NanoDrop One (Thermo Fisher Scientific, Waltham, MA, USA), cDNA was synthesized using 100 ng of RNA according to the instructions of the LunaScript^®^ RT SuperMix Kit (New England Biolabs, Ipswich, MA, USA). The relative mRNA expression levels were subsequently detected using a QuantStudio3 Real-Time PCR instrument (Thermo Fisher Scientific, Waltham, MA, USA). 40 amplification cycles of the synthesized cDNA were performed using DEPC-treated water, Luna^®^ Universal Probe qPCR Master Mix (New England Biolabs, Ipswich, MA, USA) and target gene primer/probe assays purchased from Integrated DNA Technologies (IDT, Coralville, IA, USA). The transcript levels of the target genes were normalized to the levels of the housekeeping gene GAPDH. The levels detected in the M1/M2 macrophages co-cultured with cell-free β-TCP scaffolds (for analyzing co-cultured macrophages) or in the 3DJPCs co-cultured with M1 (for analyzing co-cultured JPCs) were set as the control groups, and the relative mRNA expression levels were calculated using the 2^−(∆∆Ct)^ method.

### 4.6. Chemokine Detection in Supernatants from Co-Cultured M1/M2 Macrophages by Proteome Profiler Arrays

After co-culture of 3DJPCs/3DOBJPCs and M1/M2 macrophages for five days, supernatants from macrophage chambers were collected and stored at −80 °C. The human chemokine array kit (R&D Systems, Minneapolis, MN, USA) was used to analyze semi-quantitative secretion levels in collected supernatants. Briefly, chemokine array membranes were pretreated with a blocking buffer for one hour at room temperature and then incubated overnight at 4 °C with the mixtures of sample supernatant and antibody cocktail. After washing, membranes were incubated with diluted streptavidin-HRP for 30 min at room temperature. After additional washing, membranes were incubated with 1 mL of chemiluminescent reagent mixture and exposed to radiographic film (Cytiva, Marlborough, MA, USA) for 10 min. After scanning the developed and fixed X-ray film, positive signals were determined semi-quantitatively using the ImageJ 1.54g Java 1.8.0_345 64-bi software (NIH, Bethesda, MD, USA) and the pixel density ratio of target protein/spots to reference spots was calculated for data analysis.

### 4.7. Functional Assay: Influence of M1/M2 Secretomes on CD4^+^ T Cell Proliferation

Supernatants from M1/M2 macrophages chambers were collected after 5 days of co-culture with 3DJPCs and stored at −80 °C. After thawing, supernatants were centrifuged at 600× *g* to remove cell debris and concentrated 20-fold by centrifugation using Vivaspin 20 (Sartorius, Goettingen, Germany).

Peripheral blood mononuclear cells (PBMCs) were isolated from fresh blood of three independent donors which were collected by using monovettes with 1.6 mg EDTA/mL (SARSTEDT AG & Co. KG, Nümbrecht, Germany) by using the gradient centrifugation with Ficoll-Paque PLUS (Cytiva, Uppsala, Sweden). After extraction of the PBMC fraction and washing three times with PBS, PBMCs were used for further separation of the CD4^+^ T cells using the CD4^+^ T cell isolation kit (Miltenyi Biotec, Bergisch Gladbach, Germany) following the manufacturer’s instructions. The CD4^+^ cell fraction was stained with mouse anti-human CD4-PE (clone: M-T466, isotype: IgG1k, Miltenyi Biotec, Bergisch Gladbach, Germany) and analyzed using flow cytometry.

CD4^+^ T cells were seeded at a density of 10^6^ cells/mL in 48-well plates and cultured for 3 days in 990 µL TexMACS medium containing 5% hPL, supplemented with 20 IU/mL human IL-2 (Miltenyi Biotec, Bergisch Gladbach, Germany) and 10 µL T Cell TransACT (Miltenyi Biotec, Bergisch Gladbach, Germany) for CD4^+^ T cells stimulation. Then, activated T cells were labeled with the CFSE cell proliferation kit (Invitrogen, Waltham, MA, USA) following manufacturer recommendations. 1 × 10^5^ CFSE-labeled cells were cultured in TexMACS medium containing 5% hPL, supplemented with 20 IU/mL human IL-2 in U-bottom 96-well plates and treated with 2-fold concentrated macrophage secretomes obtained after co-culture with 3DJPCs for 72 h. At least 5000 cells of CD4^+^ T cells per condition were analyzed by flow cytometry.

### 4.8. Functional Assay: Effect of JPC Supernatants on Osteoclasts Differentiation

Supernatants from 3DJPCs chambers were collected after 5 days of co-culture with M1/M2 macrophages and stored at −80 °C. After thawing, supernatants were centrifuged at 600× *g* to remove cell debris and concentrated 20-fold by centrifugation using Vivaspin 20 (Sartorius, Goettingen, Germany).

Human osteoclasts were differentiated from monocyte precursors isolated from peripheral blood mononuclear cells (3 independent donors). After isolation, PBMCs were cultured in α-MEM (alpha minimum essential medium) containing 10% FBS, 1% penicillin/streptomycin, 1% amphotericin B at 37° and 5% CO_2_ in 75 mm^2^ flask with a density of 2.5 × 10^5^ cells/cm^2^, in the presence of 20 ng/mL M-CSF (PEPROTECH, Cranbury, NJ, USA). Medium was changed every third day. After 6–7 days in culture until the attached cells reached the desired confluency, cells were adjusted to a cell density of 5 × 10^5^ cells/mL in α-MEM medium supplemented with 20 ng/mL M-CSF and 20 ng/mL RANKL (PEPROTECH, Cranbury, NJ, USA) and 1 × 10^5^ cells were seeded per well of a 96-well plate coated with calcium phosphate (CaP). Osteoclasts precursors without osteoclastogenic stimuli served as negative control and those treated with both factors M-CSF and RANKL served as positive control. Cells were allowed to adhere overnight, and the medium was replaced containing 1-fold secretomes from 3DJPCs cultured chambers. Medium was changed every third day. Following 6 days of incubation, cells were fixed with 4% paraformaldehyde. Then, the control osteoclasts were stained for actin by AlexaFluor 546 labeled phalloidin solution (Invitrogen, Waltham, MA, USA) and for nuclei staining Hoechst 33342 was used (Promocell, Heidelberg, Germany). CaP coating was stained with 10 µM calcein (Sigma-Aldrich, Darmstadt, Germany) for fluorescent images. For the quantification of the resorbed pits, CaP coating of the plates was stained by Von Kossa staining. Therefore, the plates were incubated with 50 µL of 5% AgNO_3_ (SERVA Electrophoresis GmbH, Heidelberg, Germany) per well under UV radiation for 1 h, until the coating turned brown. The pit area was photographed and quantified using the ImageJ 1.54g Java 1.8.0_345 64-bi software (NIH, Bethesda, MD, USA).

### 4.9. Statistical Analysis

The results were expressed as mean ± standard error of the mean (mean ± SEM). A one-way analysis of variance (ANOVA), followed by Tukey’s multiple comparison tests, was used for data evaluation. All statistical analyses and visualizations were performed and visualized using the GraphPad Prism software 9.0.0 (La Jolla, CA, USA). A value of *p* < 0.05 was considered statistically significant.

## 5. Conclusions

The findings of the present study reflect different interactions between THP-1 derived M1/M2 macrophages and co-cultured 3D-cultivated JPCs. M1 macrophages seem to initially support the osteogenic differentiation of 3DJPCs. Besides inducing a M1→M2 phenotype switch, 3DJPCs reduce macrophage ability to interact with CD4^+^ T cells and inhibit osteoclast formation at the same time. Altogether, 3DJPCs possess the ability to alter macrophage plasticity.

## Figures and Tables

**Table 1:** Abbreviations, experimental groups, cells and media used for the co-culture experiments.

Abbreviation	Group	Cells	Media
TCP	β-TCP scaffold control	-	control medium
OBTCP	osteogenic β-TCP scaffold control	-	osteogenic medium
3DJPC	co-cultured untreated JPC-seeded β-TCP scaffold	JPCs	control medium
3DOBJPC	co-cultured osteogenically induced JPC-seeded β-TCP scaffold	JPCs	osteogenic medium

**Table 2:** Gene expression values (means ± SEM) of M1 macrophages after five days of co-culture with 3DJPCs/OB3DJPCs or with cell-free β-TCP scaffolds (TCP/OBTCP).

Gene	TCP-M1	3DJPC-M1	OBTCP-M1	3DOBJPC-M1
*TNF-α*	1.00 ± 0.00	0.08 ± 0.01 ^a^	1.18 ± 0.11	0.07 ± 0.01 ^b^
*CCL2*	1.03 ± 0.05	0.90 ± 0.02	1.08 ± 0.09	0.56 ± 0.04 ^b^
*CCL3*	1.01 ± 0.00	0.27 ± 0.09 ^a^	0.83 ± 0.12	0.14 ± 0.07 ^b^
*CXCL10*	1.01 ± 0.00	0.20 ± 0.03 ^a^	1.10 ± 0.27	0.18 ± 0.11 ^b^
*IL-10*	1.00 ± 0.00	1.16 ± 0.04	1.98 ± 0.09	1.65 ± 0.54
*CD163*	1.01 ± 0.00	2.21 ± 0.48 ^a^	0.57 ± 0.06	0.86 ± 0.03
*CD209*	1.01 ± 0.00	1.67 ± 0.46	0.66 ± 0.07	0.99 ± 0.23
*VEGFA*	1.01 ± 0.01	1.48 ± 0.43	1.13 ± 0.34	2.17 ± 0.56
*TGF-* *β*_1_	1.00 ± 0.00	0.72 ± 0.08	0.82 ± 0.11	0.89 ± 0.17

^a^ Significant differences were detected between the TCP-M1 and the 3DJPC-M1 groups; ^b^ significant differences were detected between the OBTCP-M1 and the 3DOBJPC-M1 groups. Groups were compared by one-way ANOVA, followed by Tukey’s multiple comparison test.

**Table 3:** Gene expression values (means ± SEM) of M2 macrophages after five days of co-culture with 3DJPCs/OB3DJPCs or with cell-free β-TCP scaffolds (TCP/OBTCP).

Gene	TCP-M2	3DJPC-M2	OBTCP-M2	3DOBJPC-M2
*TNF-α*	1.03 ± 0.00	0.66 ± 0.18	1.56 ± 0.09	1.25 ± 0.41
*CCL2*	1.00 ± 0.00	3.31 ± 0.51 ^a^	1.36 ± 0.23	2.25 ± 0.24
*CCL3*	1.00 ± 0.00	2.76 ± 0.45	1.44 ± 0.06	4.17 ± 1.23
*CXCL10*	1.06 ± 0.05	0.24 ± 0.03	3.24 ± 0.31	0.53 ± 0.20 ^b^
*IL-10*	1.01 ± 0.01	1.99 ± 0.17 ^a^	0.84 ± 0.20	1.52 ± 0.17
*CD163*	1.00 ± 0.00	8.26 ± 0.98	2.74 ± 0.12	16.96 ± 6.49
*CD209*	1.00 ± 0.00	2.54 ± 0.22 ^a^	1.66 ± 0.12	3.84 ± 0.46 ^b^
*VEGFA*	1.00 ± 0.00	5.77 ± 2.47	1.71 ± 0.07	7.59 ± 2.42
*TGF-* *β*_1_	1.02 ± 0.01	1.23 ± 0.17	1.45 ± 0.06	1.46 ± 0.21

^a^ Significant differences were detected between the TCP-M2 and the 3DJPC-M2 groups; ^b^ significant differences were detected between the OBTCP-M2 and the 3DOBJPC-M2 groups. Groups were compared by one-way ANOVA, followed by Tukey’s multiple comparison test.

**Table 4:** Pixel density ratios (means ± SEM) of chemokines detected in supernatants from co-cultured M1 macrophages.

Chemokine	TCP-M1	3DJPC-M1	OBTCP-M1	3DOBJPC-M1
CCL21	0.02 ± 0.00	0.06 ± 0.01 ^a^	0.02 ± 0.00	0.02 ± 0.00
CXCL5	0.02 ± 0.01	0.13 ± 0.03 ^a^	0.02 ± 0.01	0.09 ± 0.03
CX3CL1	0.00 ± 0.00	0.04 ± 0.00 ^a^	0.00 ± 0.00	0.02 ± 0.01 ^b^
CXCL1	0.43 ± 0.03	0.85 ± 0.11 ^a^	0.52 ± 0.08	0.57 ± 0.08
CCL14	0.01 ± 0.00	0.06 ± 0.01 ^a^	0.01 ± 0.00	0.02 ± 0.00
CCL7	0.07 ± 0.01	1.05 ± 0.09 ^a^	0.03 ± 0.01	0.89 ± 0.07 ^b^
CCL3/CCL4	0.50 ± 0.05	0.09 ± 0.01 ^a^	0.32 ± 0.06	0.01 ± 0.00 ^b^
CCL19	0.02 ± 0.00	0.01 ± 0.00	0.03 ± 0.01	0.00 ± 0.00 ^b^
CXCL12	0.14 ± 0.02	0.47 ± 0.15	0.09 ± 0.01	0.38 ± 0.10
CXCL11	0.19 ± 0.01	0.43 ± 0.15	0.09 ± 0.01	0.41 ± 0.03

^a^ Significant differences were detected between the TCP-M1 and the 3DJPC-M1 groups; ^b^ significant differences were detected between the OBTCP-M1 and the 3DOBJPC-M1 groups. Groups were compared by one-way ANOVA, followed by Tukey’s multiple comparison test.

**Table 5:** Pixel density ratios (means ± SEM) of chemokines detected in supernatants from co-cultured M2 macrophages.

Chemokine	TCP-M2	3DJPC-M2	OBTCP-M2	3DOBJPC-M2
CXCL8	0.10 ± 0.09	1.20 ± 0.35 ^a^	0.00 ± 0.00	1.18 ± 0.15 ^b^
CCL2	0.00 ± 0.00	0.94 ± 0.29 ^a^	0.00 ± 0.00	0.65 ± 0.11
CCL7	0.01 ± 0.00	0.13 ± 0.02	0.00 ± 0.00	0.33 ± 0.11 ^b^
CXCL12	0.05 ± 0.01	0.73 ± 0.07 ^a^	0.04 ± 0.01	0.47 ± 0.03 ^b^
CCL17	0.01 ± 0.00	0.03 ± 0.00 ^a^	0.00 ± 0.00	0.01 ± 0.01
CXCL16	0.02 ± 0.01	0.14 ± 0.05	0.01 ± 0.01	0.13 ± 0.02
CCL26	0.02 ± 0.01	0.25 ± 0.11	0.01 ± 0.01	0.20 ± 0.05
CXCL1	0.07 ± 0.03	0.78 ± 0.31	0.02 ± 0.01	0.74 ± 0.18
CXCL10	0.09 ± 0.04	0.21 ± 0.05	0.05 ± 0.02	0.15 ± 0.04
Midkine	0.24 ± 0.04	0.57 ± 0.12	0.22 ± 0.01	0.40 ± 0.08

^a^ Significant differences were detected between the TCP-M2 and the 3DJPC-M2 groups; ^b^ significant differences were detected between the OBTCP-M2 and the 3DOBJPC-M2 groups. Groups were compared by one-way ANOVA, followed by Tukey’s multiple comparison test.

## Data Availability

The data that support the findings of this study are available from the corresponding author upon reasonable request.

## Supplementary Materials

The following supporting information can be downloaded at: https://www.mdpi.com/article/10.3390/ijms25042355/s1.

## References

[1] Titsinides S., Agrogiannis G., Karatzas T. (2019). Bone grafting materials in dentoalveolar reconstruction: A comprehensive review. Jpn. Dent. Sci. Rev..

[2] Fernandez de Grado G., Keller L., Idoux-Gillet Y., Wagner Q., Musset A.-M., Benkirane-Jessel N., Bornert F., Offner D. (2018). Bone substitutes: A review of their characteristics, clinical use, and perspectives for large bone defects management. J. Tissue Eng..

[3] Dimitriou R., Mataliotakis G.I., Calori G.M., Giannoudis P.V. (2012). The role of barrier membranes for guided bone regeneration and restoration of large bone defects: Current experimental and clinical evidence. BMC Med..

[4] Amini A.R., Laurencin C.T., Nukavarapu S.P. (2012). Bone tissue engineering: Recent advances and challenges. Crit. Rev. Biomed. Eng..

[5] Marion N.W., Mao J.J. (2006). Mesenchymal stem cells and tissue engineering. Methods Enzymol..

[6] He F., Umrath F., von Ohle C., Reinert S., Alexander D. (2021). Analysis of the Influence of Jaw Periosteal Cells on Macrophages Phenotype Using an Innovative Horizontal Coculture System. Biomedicines.

[7] He F., Umrath F., Reinert S., Alexander D. (2021). Jaw Periosteum-Derived Mesenchymal Stem Cells Regulate THP-1-Derived Macrophage Polarization. Int. J. Mol. Sci..

[8] Brauchle E., Carvajal Berrio D., Rieger M., Schenke-Layland K., Reinert S., Alexander D. (2017). Raman spectroscopic analyses of jaw periosteal cell mineralization. Stem Cells Int..

[9] Danalache M., Kliesch S.-M., Munz M., Naros A., Reinert S., Alexander D. (2019). Quality Analysis of Minerals Formed by Jaw Periosteal Cells under Different Culture Conditions. Int. J. Mol. Sci..

[10] Hirayama D., Iida T., Nakase H. (2017). The Phagocytic Function of Macrophage-Enforcing Innate Immunity and Tissue Homeostasis. Int. J. Mol. Sci..

[11] Orecchioni M., Ghosheh Y., Pramod A.B., Ley K. (2019). Macrophage polarization: Different gene signatures in M1 (LPS+) vs. classically and M2 (LPS−) vs. alternatively activated macrophages. Front. Immunol..

[12] Mahon O.R., Browe D.C., Gonzalez-Fernandez T., Pitacco P., Whelan I.T., Von Euw S., Hobbs C., Nicolosi V., Cunningham K.T., Mills K.H.G. (2020). Nano-particle mediated M2 macrophage polarization enhances bone formation and MSC osteogenesis in an IL-10 dependent manner. Biomaterials.

[13] Edmondson R., Broglie J.J., Adcock A.F., Yang L. (2014). Three-dimensional cell culture systems and their applications in drug discovery and cell-based biosensors. Assay Drug Dev. Technol..

[14] Langhans S.A. (2018). Three-Dimensional in Vitro Cell Culture Models in Drug Discovery and Drug Repositioning. Front. Pharmacol..

[15] Thippabhotla S., Zhong C., He M. (2019). 3D cell culture stimulates the secretion of in vivo like extracellular vesicles. Sci. Rep..

[16] Kotani S., Fujita Y., Kitsugi T., Nakamura T., Yamamuro T., Ohtsuki C., Kokubo T. (1991). Bone bonding mechanism of β-tricalcium phosphate. J. Biomed. Mater. Res..

[17] Bohner M., Santoni B.L.G., Döbelin N. (2020). β-tricalcium phosphate for bone substitution: Synthesis and properties. Acta Biomater..

[18] Alvarez M.M., Liu J.C., Trujillo-de Santiago G., Cha B.H., Vishwakarma A., Ghaemmaghami A.M., Khademhosseini A. (2016). Delivery strategies to control inflammatory response: Modulating M1-M2 polarization in tissue engineering applications. J. Control. Release Off. J. Control. Release Soc..

[19] Murray P.J., Allen J.E., Biswas S.K., Fisher E.A., Gilroy D.W., Goerdt S., Gordon S., Hamilton J.A., Ivashkiv L.B., Lawrence T. (2014). Macrophage activation and polarization: Nomenclature and experimental guidelines. Immunity.

[20] Schliefsteiner C., Peinhaupt M., Kopp S., Logl J., Lang-Olip I., Hiden U., Heinemann A., Desoye G., Wadsack C. (2017). Human Placental Hofbauer Cells Maintain an Anti-inflammatory M2 Phenotype despite the Presence of Gestational Diabetes Mellitus. Front. Immunol..

[21] Durafourt B.A., Moore C.S., Zammit D.A., Johnson T.A., Zaguia F., Guiot M.C., Bar-Or A., Antel J.P. (2012). Comparison of polarization properties of human adult microglia and blood-derived macrophages. Glia.

[22] Chui R., Dorovini-Zis K. (2010). Regulation of CCL2 and CCL3 expression in human brain endothelial cells by cytokines and lipopolysaccharide. J. Neuroinflamm..

[23] Mantovani A., Sica A., Sozzani S., Allavena P., Vecchi A., Locati M. (2004). The chemokine system in diverse forms of macrophage activation and polarization. Trends Immunol..

[24] Wang N., Liang H., Zen K. (2014). Molecular mechanisms that influence the macrophage M1–M2 polarization balance. Front. Immunol..

[25] Sica A., Mantovani A. (2012). Macrophage plasticity and polarization: In vivo veritas. J. Clin. Investig..

[26] Martinez F.O., Helming L., Gordon S. (2009). Alternative activation of macrophages: An immunologic functional perspective. Annu. Rev. Immunol..

[27] Lotinun S., Kiviranta R., Matsubara T., Alzate J.A., Neff L., Luth A., Koskivirta I., Kleuser B., Vacher J., Vuorio E. (2013). Osteoclast-specific cathepsin K deletion stimulates S1P-dependent bone formation. J. Clin. Investig..

[28] Minkin C. (1982). Bone acid phosphatase: Tartrate-resistant acid phosphatase as a marker of osteoclast function. Calcif. Tissue Int..

[29] van Megen K.M., van ‘t Wout E.T., Lages Motta J., Dekker B., Nikolic T., Roep B.O. (2019). Activated Mesenchymal Stromal Cells Process and Present Antigens Regulating Adaptive Immunity. Front. Immunol..

[30] Noronha N.C., Mizukami A., Caliari-Oliveira C., Cominal J.G., Rocha J.L.M., Covas D.T., Swiech K., Malmegrim K.C.R. (2019). Priming approaches to improve the efficacy of mesenchymal stromal cell-based therapies. Stem Cell Res. Ther..

[31] Kim D.S., Jang I.K., Lee M.W., Ko Y.J., Lee D.H., Lee J.W., Sung K.W., Koo H.H., Yoo K.H. (2018). Enhanced Immunosuppressive Properties of Human Mesenchymal Stem Cells Primed by Interferon-gamma. EBioMedicine.

[32] Hughes C.E., Nibbs R.J. (2018). A guide to chemokines and their receptors. FEBS J..

[33] Xuan W., Qu Q., Zheng B., Xiong S., Fan G.H. (2015). The chemotaxis of M1 and M2 macrophages is regulated by different chemokines. J. Leukoc. Biol..

[34] Giri J., Das R., Nylen E., Chinnadurai R., Galipeau J. (2020). CCL2 and CXCL12 Derived from Mesenchymal Stromal Cells Cooperatively Polarize IL-10+ Tissue Macrophages to Mitigate Gut Injury. Cell Rep..

[35] Jordan L.A., Erlandsson M.C., Fenner B.F., Davies R., Harvey A.K., Choy E.H., Errington R., Bokarewa M.I., Williams A.S. (2018). Inhibition of CCL3 abrogated precursor cell fusion and bone erosions in human osteoclast cultures and murine collagen-induced arthritis. Rheumatology.

[36] Lin C., He H., Liu H., Li R., Chen Y., Qi Y., Jiang Q., Chen L., Zhang P., Zhang H. (2019). Tumour-associated macrophages-derived CXCL8 determines immune evasion through autonomous PD-L1 expression in gastric cancer. Gut.

[37] Mayoux M., Roller A., Pulko V., Sammicheli S., Chen S., Sum E., Jost C., Fransen M.F., Buser R.B., Kowanetz M. (2020). Dendritic cells dictate responses to PD-L1 blockade cancer immunotherapy. Sci. Transl. Med..

[38] Pandey V., Fleming-Martinez A., Bastea L., Doeppler H.R., Eisenhauer J., Le T., Edenfield B., Storz P. (2021). CXCL10/CXCR3 signaling contributes to an inflammatory microenvironment and its blockade enhances progression of murine pancreatic precancerous lesions. Elife.

[39] Cole K.E., Strick C.A., Paradis T.J., Ogborne K.T., Loetscher M., Gladue R.P., Lin W., Boyd J.G., Moser B., Wood D.E. (1998). Interferon-inducible T cell alpha chemoattractant (I-TAC): A novel non-ELR CXC chemokine with potent activity on activated T cells through selective high affinity binding to CXCR3. J. Exp. Med..

[40] Colvin R.A., Campanella G.S., Sun J., Luster A.D. (2004). Intracellular domains of CXCR3 that mediate CXCL9, CXCL10, and CXCL11 function. J. Biol. Chem..

[41] Tokunaga R., Zhang W., Naseem M., Puccini A., Berger M.D., Soni S., McSkane M., Baba H., Lenz H.J. (2018). CXCL9, CXCL10, CXCL11/CXCR3 axis for immune activation—A target for novel cancer therapy. Cancer Treat. Rev..

[42] Umrath F., Schmitt L.F., Kliesch S.M., Schille C., Geis-Gerstorfer J., Gurewitsch E., Bahrini K., Peters F., Reinert S., Alexander D. (2023). Mechanical and Functional Improvement of beta-TCP Scaffolds for Use in Bone Tissue Engineering. J. Funct. Biomater..

