# Supplementary material for: Three-Dimensionally Cultured Jaw Periosteal Cells Attenuate Macrophage Activation of CD4+ T Cells and Inhibit Osteoclastogenesis
Source: Int J Mol Sci. 2024 Feb 16;25(4):2355. doi: 10.3390/ijms25042355 (PMC10889513; doi:10.3390/ijms25042355)
Supplement: Supplementary file 1 [file ijms-25-02355-s001.zip › ijms-2799659-supplementary.pdf]

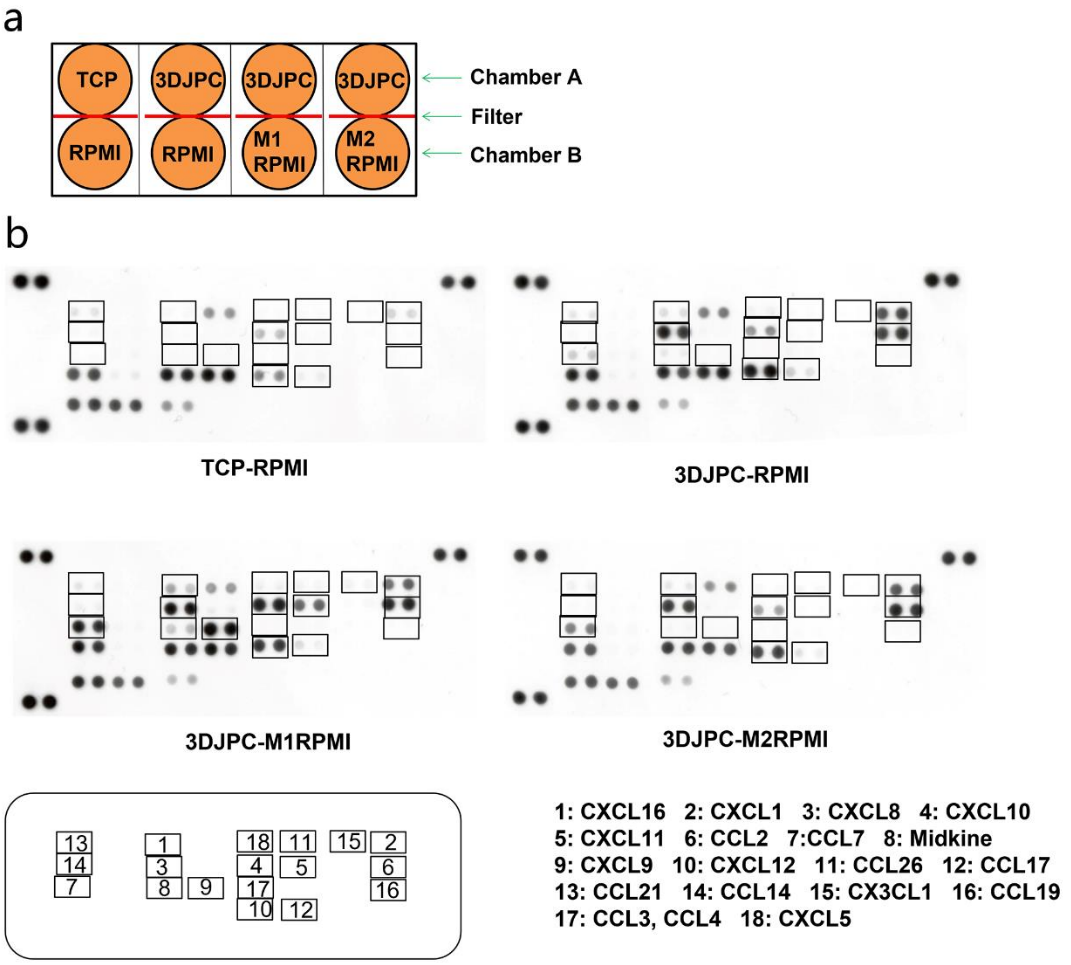


**Figure S1.** Chemokine secretion in supernatants from the 3DJPC monoculture experiment. a: Layout of the 3DJPC monoculture experiments (TCP: cell-free β-TCP scaffolds; 3DJPC: JPCs colonized β-TCP scaffolds; RPMI: cell-free RPMI complete medium; M1RPMI: cell-free RPMI complete medium + LPS + IFN-γ; M2RPMI: cell-free RPMI complete medium + IL-4 + IL-13). b: membranes with chemokine dot blots in different monoculture groups.

**Table S1.** List of chemokines detected in the supernatant of 3DJPCs monoculture experiment.

| Group | Detected in medium | JPC | LPS/IFN-γ-stimulated 3DJPC | IL-4/IL-13-stimulated 3DJPC |
|---|---|---|---|---|
| Chemokine | CXCL10 | CXCL16 | CXCL16 | CXCL16 |
| | CXCL12 | CXCL1 | CXCL1 | CXCL1 |
| | CXCL7 | CXCL10 | CXCL10 | CXCL10 |
| | CXCL4 | CXCL12 | CXCL12 | CXCL12 |
| | CCL5 | CXCL8 | CXCL8 | CXCL8 |
| | Chemerin | CCL2 | CCL2 | CCL2 |
| | CXCL1 | CCL3 | CCL3 | CCL3 |
| | CCL17 | CCL7 | CCL7 | CCL7 |
| | CCL21 | Midkine | Midkine | Midkine |
| | CCL18 | CXCL5 | CXCL5 | CXCL5 |
| | | CCL26 | CCL26 | CCL26 |
| | | CCL19 | CCL17 | CCL19 |
| | | CCL17 | CX3CL1 | CCL17 |
| | | | CCL14 | |
| | | | CXCL11 | |
| | | | CXCL9 | |
